# Collectanea Medica: Consisting of Anecdotes, Facts, Extracts, Illustrations, &c.

**Published:** 1822-05

**Authors:** 


### COLLECTANEA MEDICA:

CONSISTING OF

### ANECDOTES, FACTS, EXTRACTS, ILLUSTRATIONS, &c.

*Relating to the History or the Art of Medicine, and the Collateral Sciences.*

Floriferis, ut apes, in saltibus omnia libant,

Omnia nos, itidem, depascimur aurea dicta.

---

ART. I.—*Observations and Experiments upon the Chemical Composition of the Seeds of the Croton Tiglium, and the Oil which is procured from them.* By JOHN NIMMO, M.D. (From the Journal of Science, &c.)

ON taking a review of the various substances which have been employed, at different periods of the history of medicine, for the cure of disease, the frequency with which the opinions of medical men have changed in regard to their presumed efficacy, cannot fail to arrest the attention of the most superficial observer. In regard to many substances which have been employed during an early period of the science of medicine, the discredit into which they have fallen subsequently may be traced to the discovery of other medicinal substances, the greater efficacy of which, or convenience in administering them, has rendered the employment of preceding remedies less frequent, and at length succeeded in degrading substances which were resorted to with confidence, to that of being considered of no value whatever. Thus has hellebore fallen so low in the estimation of the medical profession, from a pre-eminent station which it once held in the cure of some of the most important diseases; most of the substances employed to produce vesication have given way, soon after cantharides was introduced into general use; thus have ipecacuanha and tartarized antimony become substitutes for other remedies; and many other instances might be adduced, from which it would appear evident, that, to other principles than caprice, alteration in the nature of diseases, and changes in the constitution of mankind arising from difference of climate or habits, must the change in opinion relative to the variable efficacy of different medicinal substances be attributed.

The seeds of the *croton tiglium* have been known to be possessed of medicinal efficacy as a purgative, nearly nine centuries. At no period, however, do they appear to have been employed generally in the practice of medicine in Europe. The disagreeable taste, excessive acrimony, and the violence of their operation, presented objections to their employment in medicine; and the danger which at times occurred in using them, produced an impression so unfavourable, that they came to be seldom used, and very soon, as if by common consent, they were banished from medical practice. Such was the state of medical knowledge relative to them, that few medical men had any practical acquaintance with them; and such as had read about them were led, by the statements which they perused, to view them as a remedy possessed of the highest degree of virulence, such, indeed, as no men could be justified in resorting to in practice without danger to their patients; or incurring the risk of injury to their professional character, by employing a remedy of so doubtful a nature. The commendations which of late have been bestowed upon the croton oil, and a sufficient quantity having been imported into this country for making trial of its efficacy, have had the effect of reviving its use in medicine; and already have many valuable communications appeared in various periodical journals, by means of which the general sense of the medical profession will soon be collected. Either it will be received into modern practice, as a powerful remedy for the cure of various diseases, or the opinions which contributed to banish it out of the field of medicine will be confirmed, and again subject it to the same general proscription which formerly it seemed so much to deserve.

A considerable time ago I procured twelve drops of this oil, for making a few trials of its efficacy, and would probably have used it in the manner recommended upon the label, but for an economical reason, —namely, to obviate the loss which would be sustained by dropping, out of a large phial, the few drops I had obtained. Recollecting the solubility of some of the fixed oils, particularly castor-oil, in alcohol, I poured upon the croton-oil two drachms of alcohol sp. gr. 825: by agitation, I perceived that a partial solution took place. Pouring this off, two drachms more of alcohol were added, by which an additional portion was dissolved. A third quantity seemed to have little effect; and, even when it was aided by heat, though a slight appearance of solution took place, the whole was precipitated when it became cold; and there was left an oily-looking substance, which appeared to be equal to somewhat more than a third part of the original quantity of oil operated upon. On tasting the alcoholic solution, I found it possessed of the characteristic acrimony of the oil: the undissolved portion had none whatever. This solution of the active ingredient of the croton-oil I considered preferable, as a medicine, to the entire oil, as obviating the following objections: namely, the difference of dose in consequence of the inequality in the thickness of the lip of phials; the greater or less degree of viscidity in the oil at different temperatures; and the difficulty of apportioning the dose to difference of age, or constitutional susceptibility to the action of the ordinary purgatives.

The rude analysis, practised above, showed that the croton-oil was not a simple substance, but a binary compound, at least: one part consisting of the purgative principle, (for, in administering the alcoholic solution in doses relative to the number of drops decomposed, the same effects were produced as have been attributed to the entire oil;) the other part inert, (for having first tasted, and then taken, the whole of the residuary oily substance, I found it entirely devoid of acrimony, slightly rancid, and possessed of no power of moving the bowels.)

The supply of croton-oil from London having been quickly exhausted, and wishing to prosecute the investigation of its properties by an extended trial, I was informed that a gentleman in town was possessed of a small quantity of the seeds, with which he was so kind as to supply me; and, with the view partly of obtaining a chemical analysis, but principally to procure some of the oil, the quantity of which in the market was reported to be nearly exhausted, the following series of experiments upon them was instituted, which, perhaps, will not be considered devoid of interest in a chemical point of view, and of utility in the medical administration of this powerful remedy.

Having weighed a quantity of the seeds, and separated the kernel from the shells, I found 100 parts contained 64 of the former and 36 of the latter. Digesting a quantity of the latter with alcohol a sufficient length of time, though a dark-coloured tincture was obtained; on tasting it, it was possessed of no acrimony whatever. It is stated, indeed, that they possess, in a considerable degree, the peculiar property of the seeds; but, in the present case, no such acrimony existed. The attention was, consequently, directed solely to the kernel of the seeds.

Taking 40 grains of the kernel, they were first reduced to a paste by bruising them in a mortar, upon which alcohol being poured, they were digested with a moderate heat several days. The whole being poured upon a filter, the fluid parts passed through; and upon the insoluble part more alcohol was poured, until every thing which was soluble in that fluid was washed away. The residuum was dried, and weighed 29 parts; 11 had been dissolved: the former tasteless, the latter resembling the alcoholic solution obtained from the entire oil. The undissolved residuary matter showed evidently that it contained a fixed oil which tinged the paper on which it was dried by heat. To discover the proportion in which this oil existed in the kernels not soluble in alcohol, it was treated with purified oil of turpentine; a substance which has not been employed in chemical analysis of vegetable substances, but which in some instances may be found advantageous.

Before, however, going on to detail the action of the purified oil of turpentine, it is necessary to remark that, in its ordinary state, it is not sufficiently pure to be employed for this or similar purposes; for, even when recently distilled, it contains a substance which, when the oil is evaporated, remains behind, and would in the present instance have led to a false estimate, by the addition of weight to the residuary undissolved substance. To remove the whole of this, I have employed alcohol to purify the oil of turpentine for medicinal use, without diminishing its efficacy, but greatly lessening its disagreeable taste and its injurious action upon the kidneys, as well as for chemical purposes. To eight parts of the oil add one part of the strongest alcohol, and let them be well agitated: in a few minutes a separation takes place; the oil, unless very impure, falls to the bottom; and the alcohol, having dissolved the impurities, floats at the top. Pour off the alcoholic portion; add an equal quantity of alcohol, as before; agitate and separate the liquids. If this be repeated three or four times, the oil becomes nearly tasteless, almost without smell, and, when a portion of it is evaporated, it leaves no residue. It is necessary to remark, that, pure as the oil may be rendered, it speedily undergoes alteration, and returns to its original state of greater or less impurity.

Upon the 29 parts of matter which remained after the action of alcohol had been exhausted, the purified oil of turpentine was poured; and, after digestion a suitable length of time, the whole was thrown upon a filter, washed with more of the oil, and the residuum thoroughly dried. Of 29 parts, there remained 16 parts undissolved, 13 parts were dissolved. These proportions being brought to 100 parts, the following is the estimate:

| | | |
|---|---|---|
| Croton seeds 100 = | 27.5 | acrid matter soluble in alcohol, |
| | 32.5 | fixed oil soluble in oil of turpentine, |
| | 40. | farinaceous matter insoluble by both. |
| | 100 | |

In another experiment, the alcoholic solution, being filtered hot, carried off 34 parts, leaving 26 for the action of the oil of turpentine, and 40 parts, just as before, undissolved. In other experiments the results were nearly in the proportions above stated.

Upon 100 parts of the bruised kernels, oil of turpentine was poured, and treated as before, yielded—

| | |
|---|---|
| 60 | parts of the acrid matter and oil soluble in it, |
| 40 | parts of insoluble farinaceous substance. |
| 100 | |

The action of sulphuric ether was next tried, and proved to be an equally good solvent of these peculiar principles with the oil of turpentine: over which, indeed, it possesses some advantages; in the view, particularly, should it ever be found necessary, of procuring the substance in the utmost degree of purity, and at no great price.

Digesting sulphuric ether upon 100 parts of the bruised seeds, throwing the whole upon a filter, covering it closely during the process of filtration, and washing the residuum with a sufficient quantity of ether, it was found to weigh 40 parts; 60 having been dissolved. By this process from 300 grains of the seeds, from which if 102 grains are deducted for the shells, there are left 198 grains of the kernels, I obtained upwards of two drachms by measure of an oil which possessed all the qualities, as to taste and medicinal efficacy, which the purchased specimen contained.

It cannot be doubted that the oil thus prepared must be as genuine as that procured by torrefaction and expression of the seeds, as practised in India; and in some respects, perhaps, it may be preferable, as tending less to occasion a decomposition of the active ingredients of the seeds, by the heat applied to effect the separation of the oily portion from the farinaceous part. A circumstance of this kind may be the cause of a considerable difference in the quality of two specimens of the oil which I have seen, in which the colour and fluidity are not the same, besides a difference in their relative strength as purgatives, without supposing the existence of any fraud on the part of those persons who have it for sale.

To determine, more accurately than by any of the former experiments, the relative proportion of the acrid purgative principle to the fixed oil, it seemed necessary to adopt the following plan:—From the first, compared with the second experiment with alcohol, it was remarked that heat rendered more of the oil soluble than when the fluid was cold; and it is obvious that the apparent quantity of oil must have been diminished by that portion which the alcohol was able to dissolve at the ordinary temperature of the atmosphere. Using a weaker alcohol, of sp. gr. .834, into which there was poured a quantity of olive oil, to which the insoluble part of the croton-oil bears the strongest resemblance; agitating, besides heating them, together, that the alcohol might become saturated with the oil: upon 40 grains (or 84 drops) of the oil, which had been extracted from the seeds by ether, this alcoholic solution of oil was poured in successive quantities. A bright yellow-coloured solution of the acrid principle was thus obtained. The insoluble portion weighed 22 grains; the dissolved part was, of course, 18 grains.

From the preceding data, the composition of the kernels of the croton is the following:

27 of acrid purgative principle,
33 fixed oil,
40 farinaceous matter.

100

The oil itself is composed of—45 acrid principle,
55 fixed oil.

100

When the alcoholic solution was dropped into solution of litmus, it turned it red, showing the presence of an acid; but, when a solution of alkali of known strength was added, the quantity was found to be so extremely small, that its nature could not be ascertained without destroying more of the oil than was in my possession. When poured into water, the mixture becomes nebulous; when passed through filtering paper, the precipitate is retained by it. The clear water which was procured from a proportion of the alcoholic solution, containing four doses of a sufficient strength to produce a purgative effect, being taken, had no effect in moving the bowels. The acrid substance appears, from this and other circumstances, to reside in a resinous principle, which is soluble in alcohol, sulphuric ether, volatile and fixed oils.

From the difference of effect which has been noticed by those who have examined the action of the croton-oil, there is room for suspecting that, in many instances, additions have been made to the real oil; and to such practices it is manifest there is a strong temptation, from the high price at which it is sold, and the facility with which adulteration can be practised without any apparent means of detection. The observations and experiments stated above, it is hoped, will be found to furnish the ready means of detection. Let a very light phial be counterpoised in an accurate balance; pour into it 50 grains, or more, of the croton-oil; add alcohol which has been digested upon olive-oil, of which it dissolves so little as not to injure, in the smallest degree, the alcoholic solution for subsequent use; agitate well; pour off the solution, and add more alcohol, in the same manner, until the dissolved portion is diffused in such a proportion of alcohol that each half-drachm measure shall contain equal to one dose of the croton-oil for an adult: by placing the phial near a fire, to evaporate what remains of the alcohol in the bottle, if the remainder be to that which has been abstracted by the alcohol as 55 to 45, the oil is genuine: if olive or any other oil, little soluble in alcohol, has been added, the residuum will be in larger proportion. But, if castor-oil has been employed, the proportion of the residue will be smaller than in the genuine medicine.

The alcoholic solution appears to me the best vehicle for administering the active principle of the croton-oil; and furnishes the means of readily proportioning the dose to the various circumstances of cases under treatment. The following is the formula which I have employed, and the directions for avoiding the uneasy feelings which are produced in the mouth and throat.

℞. Alcohol. Croton, ʒss.

Syrupi simplicis.

Mucilag. Gum. Arab. āā ʒij.

Aq. distillat. ℥ss. M.—ft. haustus.

After swallowing a little milk, take the draught very quickly, and wash it down with repeated quantities of the same diluent.

Having already administered this remedy in private practice, in considerably above an hundred doses, and to some patients many times, a copious list of cases might be furnished. At present it may be sufficient to state, that in not more than three or four cases was vomiting produced, and that not in a violent degree;—in not many more was nausea felt;—in all the cases purging was induced in a space of time between half an hour and three hours after taking the medicine. The purgative effects were generally moderate, accompanied with griping, rarely; and in proportion, generally, to the effect which was intended to be produced: circumstances which some of my medical friends, whom I furnished with the alcoholic solution prepared from the seeds, as well as myself, are inclined to attribute to the instantaneous and equable diffusion of the active principles of the croton-seeds over the inner coat of the stomach and abdominal viscera, when the above formula was employed: while, on the contrary, it must unavoidably happen that the oil, taken merely mixed with any fluid, made up into the form of pill with any substance, or diffused with sugar or starch, may at times be applied, in a concentrated form, to a particular part of the stomach or intestines, and excite an action such as to occasion nausea and vomiting in the former case, and spasmodic action, with pain and hypercatharsis, in the latter. When the unfavourable circumstances last mentioned occur, there is, no doubt, much reason to fear that this purgative may excite inflammation, or induce a state of greater or less debility. Among the cases I have had to treat was one of a lady, who, after using diuretic medicines of the most powerful kind, and undergone a course of mercurial inunction for the cure of abdominal dropsy, was rapidly sinking under an accumulation of the dropsical fluid, and nearly a total loss of appetite and strength. Her state being almost hopeless, the mixture of alcoholic solution of the croton was administered at first with extreme caution; and afterwards, when it was found she could bear it, in augmented doses, so as to cause three or four evacuations of the bowels daily, with the most beneficial consequences. By augmenting the appetite and the strength, and by the discharge of watery stools, the speedy reduction of the size of the abdomen became apparent. After two weeks' use, the irritability of the stomach made it necessary to stop the use of it. The complaint showed strong symptoms of returning: diuretics were then employed, but the effect which they had upon the appetite, and in inducing debility, soon made it necessary to desist. The croton mixture was again used; and, by employing opiates and carminatives, she was enabled to continue its use until a cure was accomplished.

In the cure of delirium tremens, I have found it a powerful auxiliary to opium. In one case of this disease, arising from excessive intemperance in the use of ardent spirits, in which the most distressing phantasms appeared, the wildest imaginations were produced, and sounds heard,—the immediate effects of a full dose of the croton solution were very apparent on one occasion, and equally so on a subsequent attack from the same cause. In another case, where similar symptoms existed, but not from the same cause, in a gentleman from the West Indies, and most probably the precursor of mania, it was equally beneficial. In this case, though a person of a very highly cultivated mind, there were present all the symptoms which have been described as arising from the belief and superstitious dread impressed on the minds of negroes in West-India plantations, from the spells and incantations of *obi*, or African witchcraft.

In all cases in which there is a superfluity of the secretion of bile regurgitating into the stomach, it is of service, unless the medicine itself is rejected by vomiting; and, in the removal of jaundice from obstruction or spasms in the bowels, (perhaps equally in those of the biliary passages,) it proved most beneficial and agreeable in its effects, as reported to me by the gentleman who furnished me with the seeds. In a case of a child, about two years of age, with almost total want of appetite, and labouring under what appeared to be an incurable state of tympanites intestinalis, it was prescribed in doses to cause two or three stools daily. In four days the child, belonging to poor parents, was brought back to me, with an appearance so much improved, in so short a period of time, as scarcely to be credible; the return of appetite, and a liveliness of countenance, and such a reduction in the tympanitic symptoms, that I formed the most sanguine hopes of ultimate recovery. Though the mother made many promises to return again, she neglected them; and, though no determinate conclusion can be drawn from the early symptoms of amendment in this case, which was truly hopeless at first view, I am induced to think that, in the cure of this most stubborn complaint, much benefit may be expected from the cautious, but steady, use of the croton-oil.

In a case of excessive corpulence, with the most alarming symptoms of determination to the head, amounting at times almost to apoplexy, a few doses of the solution produced the most signal benefit; and, without having had occasion to let blood, every symptom was removed which could have been expected from venesection; and the patient being, forthwith, put upon a regulated system of diet, exercise, and the occasional use of the croton mixture, the size of the body is slowly, but progressively, diminishing. Exercise can now be taken without hurrying the breathing,—the mind has regained its accustomed activity,—and, if the plan be steadily persisted in, there is no reason to dread that even the natural tendency, in this case, of the constitution to produce too much blood, and the deposition of an excessive quantity of fat, at the early period of thirty-two or thirty-three years, may be kept at bay, and life preserved.

**Art. III.**—*Register of Deaths, occasioned by different Species of Pectoral Diseases, compared with the general Mortality of Paris.*

| Designation of Diseases. | Years. | Number of Deaths, *in reference to the Season.* Spring. *Sex.* M. | F. | Summer. *Sex.* M. | F. | Autumn. *Sex.* M. | F. | Winter. *Sex.* M. | F. | Total *of the Annual Deaths occasioned by each of the Pulmonic Affections.* *Sex.* M. | F. | Total | Comparison *between the Number of Deaths caused by Pulmonary Diseases, and the total Number of Deaths occurring in Paris.* Total Mortality in Paris. M. | F. | Total per Year. | Proportion of the Number of Deaths referrible to each Sex. M. 1 in | F. 1 in | *Of the 2 Sexes* 1 *in* | Average proportion of the number of Deaths, referrible to each Sex, during four Years. M. 1 in | F. 1 in | *Of the 2 Sexes* 1 in |
|---|---|---|---|---|---|---|---|---|---|---|---|---|---|---|---|---|---|---|---|---|---|
| Asthma | 1816 | 39 | 35 | 13 | 14 | 17 | 20 | 43 | 56 | 112 | 129 | 237 | 9443 | 9611 | 19124 | 84 | 77 | 80.69 | | | |
| | 1817 | 22 | 24 | 12 | 13 | 14 | 24 | 34 | 30 | 82 | 91 | 173 | 10535 | 10589 | 21124 | 128 | 116 | 122.10 | 99.05 | 102.44 | 100.75 |
| | 1818 | 26 | 24 | 17 | 16 | 23 | 29 | 39 | 24 | 105 | 93 | 198 | 10770 | 11651 | 22421 | 102 | 125 | 113.24 | | | |
| | 1819 | 23 | 27 | 22 | 10 | 30 | 27 | 48 | 52 | 123 | 116 | 239 | 11054 | 11616 | 22670 | 89 | 100 | 94.85 | | | |
| | Total | 110 | 110 | 64 | 53 | 84 | 100 | 164 | 162 | 422 | 425 | 847 | 41802 | 43537 | 85339 | | | | | | |
| Average No. of Deaths, according to the Sex of the Patient. | | 27.50 | 27.50 | 16 | 13.25 | 21 | 25 | 81 | 40.50 | 105.50 | 106.25 | | 10450 | 10884 | | | | | | | |
| Of both Sexes .. | | 55 | | 29.25 | | 46 | | 81.50 | | 211.75 | | | 21334 | | | | | | | | |
| Catarrh. | 1816 | 170 | 188 | 108 | 106 | 149 | 165 | 271 | 302 | 698 | 761 | 1459 | 9443 | 9681 | 19124 | 13 | 12 | 13.10 | | | |
| | 1817 | 231 | 225 | 107 | 115 | 177 | 182 | 169 | 154 | 684 | 676 | 1360 | 10535 | 10589 | 21124 | 15 | 15 | 15.53 | 15.29 | 14.05 | 14.63 |
| | 1818 | 187 | 238 | 107 | 131 | 131 | 178 | 238 | 221 | 663 | 768 | 1431 | 10770 | 11651 | 22421 | 16 | 15 | 15.67 | | | |
| | 1819 | 188 | 253 | 87 | 139 | 161 | 246 | 253 | 256 | 689 | 894 | 1583 | 11054 | 11616 | 22670 | 16 | 12 | 14.32 | | | |
| | Total | 776 | 904 | 409 | 491 | 618 | 771 | 931 | 933 | 2734 | 3099 | 5833 | 41802 | 43537 | 85339 | | | | | | |
| Average according to the Sex of the Patient | | 194 | 226 | 102.25 | 122.75 | 154.50 | 193.75 | 232.75 | 233.75 | 683.50 | 774.75 | | 10450 | 10884 | | | | | | | |
| Of both Sexes .. | | 420 | | 225 | | 347.25 | | 466 | | 1458.25 | | | 21334 | | | | | | | | |

| | | | | | | | | | | | | | | | | | | | | |
|---|---|---|---|---|---|---|---|---|---|---|---|---|---|---|---|---|---|---|---|---|
| PULMONIC INFLAMMATION. 1816 | 114 | 82 | 42 | 30 | 56 | 60 | 129 | 108 | 341 | 286 | 627 | 9443 | 9681 | 19124 | 27 | 33 | 30.50 | | | |
| 1817 | 132 | 126 | 51 | 27 | 42 | 40 | 89 | 66 | 314 | 259 | 573 | 10535 | 10589 | 21124 | 33 | 40 | 36.87 | 30.11 | 32.93 | 31.94 |
| 1818 | 85 | 106 | 68 | 70 | 94 | 73 | 80 | 118 | 327 | 367 | 694 | 10770 | 11651 | 22421 | 33 | 32 | 32.56 | | | |
| 1819 | 129 | 100 | 52 | 51 | 84 | 107 | 141 | 152 | 406 | 410 | 816 | 10052 | 11616 | 22670 | 27 | 28 | 27.78 | | | |
| Total | 460 | 420 | 213 | 178 | 276 | 280 | 439 | 444 | 1388 | 1322 | 2710 | 41802 | 43537 | 85339 | | | | | | |
| Average according to the Sex of the Patient | 115 | 105 | 53.25 | 44.50 | 69 | 70 | 109.75 | 111 | 347 | 330.50 | | 10450 | 10884 | | | | | | | |
| Of both Sexes .. | 220 | | 97.75 | | 139 | | 220.75 | | 677.50 | | | 21334 | | | | | | | | |
| PHTHISIS. 1816 | 227 | 398 | 206 | 308 | 204 | 315 | 248 | 360 | 885 | 1381 | 2266 | 9443 | 9681 | 19124 | 10 | 7 | 8.43 | | | |
| 1817 | 288 | 363 | 254 | 338 | 278 | 302 | 260 | 298 | 1080 | 1301 | 2381 | 10535 | 10589 | 21124 | 9 | 8 | 8.88 | 10.54 | 7.80 | 8.49 |
| 1818 | 229 | 389 | 222 | 293 | 237 | 379 | 245 | 324 | 933 | 1385 | 2318 | 10770 | 11651 | 22421 | 12 | 8 | 9.67 | | | |
| 1819 | 306 | 440 | 237 | 366 | 243 | 332 | 281 | 372 | 1067 | 1510 | 2577 | 10052 | 11616 | 22670 | 10 | 7 | 8.78 | | | |
| Total | 1050 | 1590 | 919 | 1305 | 962 | 1328 | 1034 | 1354 | 3965 | 1577 | 9542 | 41802 | 43537 | 85339 | | | | | | |
| Average according to the Sex of the Patient | 262.50 | 397.50 | 288.50 | 326.50 | 240.50 | 332 | 258.50 | 338.50 | 991,25 | 1394.25 | | 10450 | 10884 | | | | | | | |
| Of both Sexes .. | 660 | | 554.75 | | 572.50 | | 597 | | 2385.50 | | | 21334 | | | | | | | | |
| Recapitulation of the four Diseases | | | | | | | | | | | | | | | | | | | | |
| Total | 2396 | 3024 | 1605 | 2027 | 1940 | 2479 | 2568 | 2893 | 8509 | 10423 | 18932 | 41802 | 43537 | 85339 | | | | | | |
| Average No. of Deaths .... | 599 | 756 | 401 | 507 | 845 | 619 | 642 | 723 | 2127 | 2606 | | 10450 | 10884 | | | | | 4.91 | 4.17 | 4.52 |
| and Both Sexes...... | 1355 | | 908 | | 1104 | | 1365 | | 4733 | | | 21334 | | | | | | | | |

### Art. III.—*Division of the Pneumogastric Nerves, and supposed Influence of Galvanism on Digestion.*

When the question, involved in the above title, was keenly agitated, we expressed, in another publication, our opinion of its puerile and unphysiological nature. We had, then, on our side, facts, analogy, strict anatomical inferences, the Royal Society, Messrs. Brodie, Magendie, and Broughton, and a score more of persons who, accustomed to experimental inquiries, could not but see the absurdity of the doctrine attempted to be palmed on the English public. On the other side stood the promulgator of the new doctrine, Dr. W. Philip, and its only supporter, Dr. Hastings.

Those who take any interest in these passing bubbles of the day, will recollect how soon Dr. W. Philip's conclusions, deduced from insulated experiments, were controverted, and their results found to be incorrect. Dr. Hastings, however, having endeavoured to refute the mass of evidence brought against his friend's theory, a fresh trial of the question was determined upon, in the presence of each contending party; when, Dr. Philip informs us, even Mr. Brodie himself became satisfied that the nervous influence performed all the wonders which Dr. Philip had ascribed to it.

In answer to this last statement, we cannot do better than to bring forward the reflections of a very sensible writer in the last Number of the Edinburgh Medical Journal, containing an exposition of the fallacy of Dr. Philip's premises, as well as of his conclusions. Should the parties, after the perusal of the arguments used by the writer in question, still feel inclined to carry on their unprofitable controversy, we must express our hopes that they will be induced to embody their lucubrations in some *ex professo* pamphlet, to be bought or not, just as the whim of the moment may suggest, rather than attempt to force them down the throat of each professional reader, *volente*, *nolente*, by encumbering with them every valuable periodical Journal in the metropolis. A. B. G.

---

After the specimen of scientific dissent and agreement which we have here exhibited, it might be deemed truly presumptuous in us to offer any opinion of our own, or even to assume the part of mediators; and indeed the discordant results of the trials made by experimentalists offer no encouragement to future researches. But the few experiments of the kind which we have seen,—the effects of those which we have known,—and the little knowledge of the nerve concerned, to which we may, without excessive arrogance, lay claim,—have suggested some reflections which we are unwilling to withhold.

1st. We think it is quite certain that the division of the pneumogastric nerve, which Dr. W. P. has employed, is entirely unsuitable to demonstrate the influence which this operation exercises on the stomach, its actions, or secretions. The experimental researches of M. Le Gallois, and many authors both before and after him, have clearly shown that the most conspicuous effect arising from this operation, was referrible to the paralysis of the arytenoid muscles, and the consequent pulmonary congestion and unhealthy bronchial secretion. We must, therefore, lay it down as a settled point, that it is extremely difficult to analyze the various effects resulting from this operation, and to separate and estimate those which are truly referrible to lesion of the actions and functions of the stomach. We entirely agree with M. Magendie in thinking that, in order to determine what is the peculiar effect resulting from the operation, it is requisite to divide those twigs or fibrils only of this nerve which go to the stomach.* This operation has been, indeed, performed by Dupuytren, Magendie, Brodie, &c. and the effects have not accorded with those which Dr. Wilson Philip's doctrines would lead us to anticipate; but a great objection to the facility of observing and drawing legitimate and direct inferences, is the injury necessarily done to the animal in the operation; and we are almost certain that few logical or unbiassed inquirers would be disposed to draw conclusions from the present scanty, discordant, and uncertain, results which are related by various experimentalists.

2d. We cannot divine what has tempted Dr. W. Philip to fix on this unfortunate eighth pair for all his doctrines on the subject of digestion, and especially that most mysterious of all organic processes, secretion. Why does he render it the exclusive and individual agent of so many important phenomena? It is certainly not the only gastric nerve, and it is subservient to many other purposes besides sending a few twigs to the stomach. It is unnecessary, and would be perhaps nugatory, to quote our own authority on a matter of fact and observation, in which every one observer must be nearly as well informed as another; but, if we adduce the authority of Walter, &c. to support our own, we find that we are correct in saying that the gastric twigs of the right *nervus vagus* are found contiguous, chiefly, to the pyloric region, and thence along the small curvature, in the course of which they communicate with those of the left side; that the gastric twigs of the left *nervus vagus*, proceeding from the cardiac region along the small curvature, descend obliquely, in general with ramifications of the coronary artery, and either sink into the substance of the gastric tissues, or form anastomoses with the twigs of the left branch. Throughout this entire course, it forms repeated and innumerable connexions with the splanchnic nerves, many divisions of which go to the gastric tissues, in company with the right and left gastro-epiploic arteries, along the convex or great curvature of the stomach. The reflections to which this anatomical distribution must give rise, did not escape Dr. Haighton; but, if we are again to be perplexed with this everlasting wandering nerve, why are we not informed of the effects which arise from the division of its gastric twigs only, and of the division of the other gastric nerves,—as, for example, the splanchnic branches? However we may respect the physiological zeal and the indefatigable enthusiasm of Dr. W. Philip, still it is impossible to admit the inferences which he has derived from the division of the pneumo-gastric nerves, such as he has practised it.

* *Précis Elementaire*, tom. ii. p. 94, 95.

3d. On the second part of Dr. W. P.'s doctrine, the analogy between the galvanic and the nervous power or action, we are not favoured with any observations from either of the parties;—Mr. Broughton, in the early part of his researches, deeming such inquiry unnecessary, and subsequently relating, as we have seen, what was observed in the presence of himself and Dr. W. P.; and Dr. Hastings declining to enter on the subject at all. We must admit our inability to understand precisely whether Dr. W. P. admits the existence of an actual nervous influence, and its analogy to the galvanic action; or whether he means to say, that the galvanic action merely excites the nervous and the muscular system, or the extremities of arteries concerned in the process of secretion. If the former be his opinion, he is certainly almost the only physiological writer of any eminence who espouses such a puerile and unfounded speculation, and who has the weakness to explain physiological phenomena by referring them to physical ones equally obscure and unintelligible.

From his language, in the concluding sentence above quoted, it may be inferred that he deems this nervous influence an actual material existence, of a palpable nature, and which is conveyed through sensible distances by the animal tissues. Is it quite consistent, we would ask, to consider this influence as capable of being conveyed, in the one case over the space of one-sixth of an inch, in the other one-fourth of an inch, not through nervous texture, but along the contiguous cellular membrane, or, as it may happen, through the serous or bloody fluid interposed between the two cut extremities; and then to inform us that, if the lower portion be folded back, (which can make only a very trifling difference,) this influence no longer passes to that inferior portion, which receives and transmits the galvanic action only? What, in this case, becomes of the influence transmitted from the superior portion of the nerve? Can it not be made to pass a little further,—for example, one or two lines,—and then enter the lower portion by the side? or can it not make a trifling circuit, and, with a sort of secret affinity for its natural organic conveyance, enter even by the folded extremity, as well as by its direct route? It might be concluded, from the very manageable nature of the nervous influence in the hands of our physiologist, that all these things might have been accomplished without much difficulty.

On Dr. W. Philip's supposition that this nervous influence possesses no power, sensorial or vital, but that of a chemical agent, we feel it difficult either to form or deliver an opinion; because that opinion, at present, whatever may subsequently induce us to change it, can express only dissent from the conclusions which he has formed.

It is surely not in the nineteenth century, after Stahl, Cullen, Hunter, and Bichat, have passed successively before the eyes of their fellow-men;—it is surely not in the nineteenth century, after Newton, D'Alembert, and La Place, have shown what physical properties can do, and what is beyond their reach,—that physicians are to be amused with this galvanic hypothesis of nervous influence and secretion: nor are we to be informed, by those who seem so little conversant with the peculiar nature of chemical and vital phenomena, that the duties of this supposed nervous influence, or energy, may be performed by the agency of the voltaic pile. We cannot deny that the galvanic power excites the muscular fibre like any other agent, chemical or mechanical, and may possibly, in the same manner, maintain or increase the gastric secretions: but this neither proves its analogy with the properties of the nervous system, nor enables us to perceive in what manner secretion and muscular action are performed. We must presume that Dr. W. Philip and Dr. Hastings are aware of these distinctions; yet we are not told whether any means have been employed to obviate or avoid fallacies of this description. We may further remark, that, in trying the effects of the galvanic action, many seem to overlook the important fact, that a nerve, or a nervous twig, does not receive or communicate the galvanic action better than any other tissue;—that a muscular fibre, or even a moistened thread, does it quite as perfectly; —and that the nerve conveys it, not because it is a nerve, but merely because it is an animal substance well moistened, and continuous with the part to be excited. The practice of applying galvanism to nerves is indeed a remnant of that mystical physiology which, centuries ago, led Paracelsus to dream of his radical heat and moisture, and Stahl and Van Helmont of their vital principle; and to which we generally remark, that all those who, in the present day, aspire at an extraordinary character for researches in those obscure and shadowy regions of science, to which they are sure few will follow to expose their errors, never fail to manifest an inclination. We do not pretend to have smitten to the death so many rabbits and dogs as these experimentalists have very laudably done; but this does not prevent us from perceiving, that not only have they misled others by their inferences, but they have also deceived themselves in their results. We will neither ridicule nor censure this mode of unfolding the secrets of nature in regard to the animal economy; for it is one to which we owe much, and from which, if properly directed, we may yet hope to derive infinite assistance. But the facility with which most experimentalists render the results of their researches subservient to their own opinions exclusively, is an objection which will prevent either party from yielding to the other, and cannot fail to darken and perplex any subject which is, of itself, neither clear nor obvious.

This is, indeed, one of those felicitously ambiguous subjects, in which men may long dispute without either comprehending each other, or perceiving the precise nature of the points on which they are at issue; and all those who are interested in the progress of rational and sober physiology would wish it to be either investigated in a manner totally different from that in which it has been hitherto done, or entirely banished from the confines of a science which ought to rest on no other foundation but that of accurate observation, and experiment unsophisticated by hypothesis.—*Edinburgh Med. Journal.*

